# ATM-Dependent Phosphorylation of Hepatitis B Core Protein in Response to Genotoxic Stress

**DOI:** 10.3390/v13122438

**Published:** 2021-12-05

**Authors:** Barbora Lubyova, Eva Tikalova, Kristyna Krulova, Jan Hodek, Ales Zabransky, Ivan Hirsch, Jan Weber

**Affiliations:** 1 IOCB Gilead Research Center, Institute of Organic Chemistry and Biochemistry of the Czech Academy of Sciences, 160 00 Prague, Czech Republic; eva.tikalova@uochb.cas.cz (E.T.); kristyna.krulova@uochb.cas.cz (K.K.); jan.hodek@uochb.cas.cz (J.H.); ales.zabransky@uochb.cas.cz (A.Z.); hirschi@natur.cuni.cz (I.H.); 2 Department of Genetics and Microbiology, Faculty of Science, Charles University, BIOCEV, 252 50 Vestec, Czech Republic

**Keywords:** HBV core protein, serine phosphorylation, DNA damage response pathway, ATM, ATR

## Abstract

Chronic hepatitis caused by infection with the Hepatitis B virus is a life-threatening condition. In fact, 1 million people die annually due to liver cirrhosis or hepatocellular carcinoma. Recently, several studies demonstrated a molecular connection between the host DNA damage response (DDR) pathway and HBV replication and reactivation. Here, we investigated the role of Ataxia-telangiectasia-mutated (ATM) and Ataxia telangiectasia and Rad3-related (ATR) PI3-kinases in phosphorylation of the HBV core protein (HBc). We determined that treatment of HBc-expressing hepatocytes with genotoxic agents, e.g., etoposide or hydrogen peroxide, activated the host ATM-Chk2 pathway, as determined by increased phosphorylation of ATM at Ser1981 and Chk2 at Thr68. The activation of ATM led, in turn, to increased phosphorylation of cytoplasmic HBc at serine-glutamine (SQ) motifs located in its C-terminal domain. Conversely, down-regulation of ATM using ATM-specific siRNAs or inhibitor effectively reduced etoposide-induced HBc phosphorylation. Detailed mutation analysis of S-to-A HBc mutants revealed that S170 (S168 in a 183-aa HBc variant) is the primary site targeted by ATM-regulated phosphorylation. Interestingly, mutation of two major phosphorylation sites involving serines at positions 157 and 164 (S155 and S162 in a 183-aa HBc variant) resulted in decreased etoposide-induced phosphorylation, suggesting that the priming phosphorylation at these serine-proline (SP) sites is vital for efficient phosphorylation of SQ motifs. Notably, the mutation of S172 (S170 in a 183-aa HBc variant) had the opposite effect and resulted in massively up-regulated phosphorylation of HBc, particularly at S170. Etoposide treatment of HBV infected HepG2-NTCP cells led to increased levels of secreted HBe antigen and intracellular HBc protein. Together, our studies identified HBc as a substrate for ATM-mediated phosphorylation and mapped the phosphorylation sites. The increased expression of HBc and HBe antigens in response to genotoxic stress supports the idea that the ATM pathway may provide growth advantage to the replicating virus.

## 1. Introduction

Chronic hepatitis (CHB) caused by the Hepatitis B virus (HBV) is a serious liver disease. CHB is associated with severe liver conditions ranging from fibrosis and cirrhosis to hepatocellular carcinoma or acute liver failure [1,2]. The World Health Organization (WHO) estimates that in 2019, 296 million people worldwide were chronically infected with HBV.

The Hepatitis B virus is a small, enveloped virus with a 3.2 kb long, partially double-stranded circular DNA genome. It encodes only four overlapping open reading frames that are transcribed into 6 RNAs and translated to seven proteins, e.g., pre-core (HBeAg), core (HBc, p21), viral polymerase (P), envelope/surface proteins (S, M and L) and the X protein (HBx) [3,4,5].

The HBV core protein is a phospho-protein consisting of 183 or 185 amino acids (aa) with an approximate molecular mass of 21.5 kDa. As demonstrated by numerous reports, the HBV core protein (HBc), which is the main component of viral nucleocapsid, plays multiple roles in the HBV life cycle [6]. In the nucleus, it has been suggested that HBc regulates transcription of HBV RNAs through direct binding to the cccDNA minichromosome as well as recruiting cellular histone acetyltransferases [7]. In the cytoplasm, HBc is a main structural component of the HBV nucleocapsid, and it participates in RNA encapsidation, reverse transcription, nucleocapsid assembly and viral release [8].

In terms of its structure, the HBc protein could be divided into N- and C-terminal domains that are connected with a 9 amino acids linker region. The N-terminal domain (NTD), also known as the assembly domain, encompasses amino acids from 1 to 140, and is necessary for capsid assembly [9,10]. The C-terminal domain (CTD), spanning amino acids from 150 to 185 (or 183), consists of four arginine-rich domains (ARDs) and is responsible for trafficking of the capsid into the nucleus and displays DNA/RNA binding and chaperone activities [11,12,13]. The CTD also contains seven serine and one threonine residues that can be phosphorylated. The balance between negatively charged phosphoryl groups and positively charged arginine residues is essential for the proper regulation of the HBV replication cycle [14,15]. The highly dynamic process of phosphorylation and dephosphorylation governs the homeostatic charge that is important for capsid assembly, pgRNA packaging and reverse transcription of pgRNA into rcDNA [6].

The major phosphorylation of the CTD occurs on each of three serine-proline (SP) sites (serines 157, 164, 172 in a 185-aa HBc variant) [16,17,18]. Phosphorylation of these major phosphorylation sites is facilitated by several host kinases, e.g., SRPK1/2, CDK2, or PKC and PKA [16,18,19,20,21]. Aside from these sites, additional minor phospho-acceptor sites in the CTD have been identified. Among them, serines 170 and 178 (in a 185-aa HBc variant), part of the serine-glutamine (SQ) motifs, were shown to be phosphorylated by polo-like kinase 1 (Plk1), leading to increased HBV replication [22]. Apart from phosphorylation, other PTMs, including arginine methylation or ubiquitin-like modification, were also recently shown in our previous studies to play an important role in HBc protein function and subcellular localization [23,24,25].

Recently, there is growing evidence that the ATM (Ataxia-telangiectasia-mutated) and ATR (ataxia telangiectasia and Rad3-related) pathways play an important role in the positive regulation of HBV replication. ATM and ATR belong to the class-IV phosphoinositide 3-kinase (PI3K)-related kinase (PIKK) family, along with the mammalian target of rapamycin (mTOR) and DNA-dependent protein kinase (DNA-PK) [26,27]. They are key regulators of the DNA damage response (DDR), and they maintain the genome integrity in eukaryotic cells. Activated ATM and ATR phosphorylate their downstream substrates, Checkpoint kinase 2 and 1 (Chk2, Chk1), respectively [28], which affects different cellular pathways, including cell cycle checkpoints and gene transcription [29]. The typical substrates for ATM and ATR are proteins with Ser or Thr residues that are followed by Gln (SQ or TQ motifs) [30].

Several studies have already documented the importance of the DDR pathway in HBV infection and replication. Zhao et al. [31,32] showed that the HBV infection activated the ATR checkpoint pathway, and led to increased phosphorylation of multiple downstream targets, including Chk1, p53 and H2AX. In another study by Kim et al. [33], cytoplasmic HBx induced reactive oxygen species (ROS) production that led to the accumulation of γ-H2AX foci and increased levels of phosphorylated Chk2 (p-Chk2). This activated ATM-Chk2 pathway led to a delay in cell cycle progression at the G2/M phase. Treatment with DNA damaging agents, doxorubicin, or hydrogen peroxide upregulated transcription of ATM and ATR that, in turn, activated HBV replication [34]. Conversely, down-regulation of ATM and ATR expression was shown to reduce levels of HBV RNAs and both intracellular and extracellular HBV DNA. Furthermore, Luo et al. [35] implicated the ATR-Chk1 pathway in control of rcDNA processing during its conversion to cccDNA. Expression knockdown of ATR and its downstream signaling factor, Chk1, decreased cccDNA formation during de novo HBV infection.

In the present study, we addressed the potential role of ATM/ATR kinases in phosphorylation of the HBc protein. The HBc protein contains three SQ motifs, all located in its C-terminal domain. Treatment of HBc-expressing human hepatocytes with DNA damaging agents, etoposide and H_2_O_2_, and subsequent mutational analysis revealed that HBc is phosphorylated in response to ATM-Chk2 activation predominantly on serine residue 170 (S168 in a 183-aa HBc variant). Importantly, etoposide treatment of HBV-infected hepatocytes led to increased levels of intracellular HBc and HBe antigen in cell supernatants as well as higher levels of HBc phosphorylation at SQ sites.

## 2. Materials and Methods

### 2.1. Cell Lines and Culture Conditions

HepG2-NTCP (a human liver cancer cell line, HepG2, stably transfected with the human HBV entry receptor sodium taurocholate co-transporting polypeptide (NTCP) was obtained from Dr. Stephan Urban (Heidelberg University Hospital, Heidelberg, Germany)). HepG2.2.15 (a HepG2 cell line that harbors two head-to-tail dimers of the HBV genome (subtype ayw, genotype D; GenBank accession: U95551.1) was obtained from Dr. David Durantel (Cancer Research Center of Lyon, Lyon, France)). They were grown in Dulbecco’s modified Eagle’s medium supplemented with 10% FBS and puromycin (0.05 mg/mL) or G418 (0.4 mg/mL), respectively.

### 2.2. Plasmids

The full-length HBc (1–185 aa, HBV genotype A, subtype adw2) expression plasmid was generated by PCR amplification of HBc ORF (as a template we used plasmid pHY92CMV obtained from Dr. Huiling Yang (Gilead Sciences, Inc., Foster City, CA, USA)), followed by subcloning into pcDNA3.1 (ThermoFisher Scientific, Waltham, MA, USA). The full-length His-HA-HBc (1–185 aa, HBV genotype A, subtype adw2) expression plasmid was constructed as follows: HBc coding sequence was amplified by PCR using primer pair HBc-F: 5′-aaacgggccctctagATGCACCATCACCACCATCACGCGAAAGAAAACCTGTATTTTCAGGGTAGC*TACCCATACGATGTTCCAGATTACGCT*GACATTGACCCGTATAAAGAATTTGG-3′ and HBc-R: 5′-gccgctcgagtctagTTAACATTGAGATTCCCGAGATTGAGATCTTCTG-3′ and Q5 polymerase (NEB, Ipswich, MA, USA) from plasmid pHY92CMV as a template. The His6x tag sequence is underlined, the HA sequence is in italics, the sequence in small capitals represents homologous regions to the *Xho*I linearized pcDNA3.1 vector. PCR product was inserted into *Xho*I linearized pcDNA3.1(-) (ThermoFisher Scientific, Waltham, MA, USA) by an In-Fusion approach (Takara Bio Inc., Shiga, Japan). The HBx expression plasmid (HBV genotype D, subtype ayw) was generated by PCR amplification of HBx ORF with primers X-Nhe-F 5′-ATATACGCTAGCACCATGGCTGCTAGGCTGTGCTGCCAAC-3′ and X-TAA-Xho-R 5′-CTCTAGACTCGAGTTAGGCAGAGGTGAAAAAG-3′ (as a template we used plasmid pUC57-HBV4.1 obtained from Dr. Huiling Yang (Gilead Sciences, Inc., Foster City, CA, USA)), followed by subcloning into the pcDNA4-V5-HisA vector. The C-terminal truncation of HBc (HBc-ΔC-HA, aa 1–149) was described previously [24]. The S-to-A mutants of His-HA-HBc were generated by PCR, using QuickChange XL Site-Directed Mutagenesis (Agilent Technologies, Santa Clara, CA, USA). The fidelity of all constructs was verified by Sanger sequencing (Eurofins Genomics, Ebersberg, Germany).

### 2.3. Antibodies

The primary antibodies and reagents used for the Western blot and immunoprecipitations include antibodies against β-actin (Abcam, Cambridge, UK), HA (Merck Millipore, Burlington, MA, USA), alpha-tubulin (Thermo Fisher Scientific, Waltham, MA, USA), lamin A/C (Santa Cruz Biotechnologies, Dallas, TX, USA) and anti-HA magnetic beads (Thermo Fisher Scientific, Waltham, MA, USA). Antibodies against HBc (rabbit monoclonal) and HBx (mouse monoclonal) were obtained from Dr. Rudolf K. Beran (Gilead Sciences, Inc., Foster City, CA, USA). The following antibodies were purchased from Cell Signaling (Danvers, MA, USA): anti-phospho-ATM/ATR Substrate (S*Q) detecting phosphorylated SQ motifs (p-SQ), anti-ATM, anti-phospho-ATM (p-ATM, Ser1981), anti-ATR, anti-phospho-ATR (p-ATR, Thr1989), anti-Chk1, anti-phospho-Chk1 (p-Chk1, Ser345), anti-Chk2 and anti-phospho-Chk2 (p-Chk2, Thr68). Secondary antibodies include goat polyclonal anti-mouse horseradish peroxidase (HRP) (Merck Millipore, Burlington, MA, USA) and goat polyclonal anti-rabbit-HRP (Merck Millipore, Burlington, MA, USA). For visualization, we used a SuperSignal^TM^ West Femto Maximum Sensitivity Substrate (Thermo Fisher Scientific, Waltham, MA, USA) and LAS-4000 imager. As secondary antibodies for the LI-COR system, we used goat anti-rabbit IgG (H + L) IRDye 800CW (LI-COR Biosciences, Lincoln, NE, USA) and goat anti-mouse IgG (H + L) IRDye 680RD (LI-COR Biosciences, Lincoln, NE, USA). The Western blots were visualized using an LI-COR Odyssey CLx system and the Image Studio Lite Software was used for densitometric analysis.

### 2.4. SiRNAs

All siRNAs were purchased from Thermo Fisher Scientific (Waltham, MA, USA). The siRNAs were s42813 and s57219 for ATM, and s536 for ATR. Two different Select Negative Control siRNAs 1 and 2 were used as the control. The siRNAs were transfected using Lipofectamine RNAiMAX (Thermo Fisher Scientific, Waltham, MA, USA), according to the manufacturer’s recommendations. The specificity and inhibitory potential of ATM- and ATR-siRNAs was estimated by RT-qPCR using the following primer pairs: ATM-F 5′-AGTGGGACCATTGCACTTCC-3′, ATM-R 5′-TCTTCCACTTCTTTTACTCTGGCA-3′; ATR-F 5′-ACTGCAGCTATCTTCCACTACC-3′, ATR-R 5′-AGTGCTCTCAGAGGTCTCCTTT-3′. RNA was isolated from transfected HepG2-NTCP cells using RNeasy Plus kit (Qiagen, Hilden, Germany) and reverse-transcribed using a Maxima First Strand cDNA Synthesis kit (Thermo Fisher Scientific, Waltham, MA, USA), according to the manufacturer’s recommendations. qPCR was performed with gb Elite PCR Master Mix (Generi Biotech, Hradec Kralove, Czech Republic) and SYBR Green dye (Thermo Fisher Scientific, Waltham, MA, USA). The 20 μL qPCR mixture consisted of 5 μL cDNA, primers (0.2 μM each), SYBR Green dye diluted 1:2500 and 1 × gb Elite Master Mix. Thermal cycling conditions for qPCR run were as follows: denaturation (95 °C for 10 min), denaturation and annealing/elongation steps repeated 40 times with fluorescence measurement at the end of the elongation step (95 °C for 15 s, 60 °C for 1 min). The levels of ATM and ATR mRNAs were normalized to housekeeping gene Hypoxanthine phosphoribosyl-transferase 1 (HPRT1). For amplification of HPRT1 cDNA, the following primers were used: HPRT1-F 5′-TGGTCAGGCAGTATAATCCAAAG-3′, HPRT1-R 5′-TTTCAAATCCAACAAAGTCTGGC-3′.

### 2.5. Chemicals and Inhibitors

For the induction of DNA damage, the cells were exposed to a 1 h treatment of etoposide (Etp), hydrogen peroxide (H_2_O_2_) or 4-Hydroxynonenal (4-HNE), all from Merck Millipore (Burlington, MA, USA). After treatment, the cells were washed twice and cultured in complete DMEM medium supplemented with 10% FBS for an additional 4 h. Dimethyl-sulfoxide (DMSO) was used as the vehicle for etoposide and 4-HNE. Both the ATM inhibitor KU55933 (Merck Millipore, Burlington, MA, USA) and ATR inhibitor IV VE821 (Merck Millipore, Burlington, MA, USA) were used at a concentration of 10 μM for two hours before etoposide treatment, and at a concentration of 5 μM after treatment.

### 2.6. HBV Infection of HepG2-NTCP Cells

Two days before infection, the cells were incubated in medium supplemented with 2.5% DMSO. The cells were infected with HepG2.2.15-derived HBV (MOI 500 viral genome equivalents (VGE) per cell) in the presence of 4% PEG8000 overnight. Sixteen hours later, the cells were washed 3 times with PBS followed by treatment with etoposide for 1 h. After treatment, the cells were washed twice and maintained in DMEM supplemented with 2.5% DMSO. At different time-points after the addition of Etp, the cells were harvested and protein extracts were isolated and analyzed by Western blotting. The levels of HBe antigens were determined from cell supernatants by ELISA at each time-point.

### 2.7. Transfections

All transfections of expression plasmids were carried out using Lipofectamine 3000 transfection reagent (ThermoFisher Scientific, Waltham, MA, USA), according to the manufacturer’s recommendations. The HepG2-NTCP cells were transfected (in 24-well plates, 6-well plates or T75 flask format) with the HBc, HBx expression plasmids, or an empty vector, pcDNA.

### 2.8. Preparation of Protein Samples

At different time-points after etoposide- or H_2_O_2_-treatment, the cells were lysed in lysis buffer (20 mM HEPES (pH 7.9), 50 mM NaCl, 5 mM EDTA, 1% (*v*/*v*) Igepal CA-630, 10% (*v*/*v*) glycerol, 1 mM dithiothreitol, 1 mM phenyl-methyl-sulfonyl fluoride (PMSF), 0.2 mM protease inhibitor cocktail (all Merck Millipore, Burlington, MA, USA)) and phosphatase-inhibitor-mix I (Serva, Heidelberg, Germany). Protein extracts were analyzed either by Western blotting or immunoprecipitation (IP). For IP, the protein extracts (800 μg or 1 mg) were incubated with anti-HA (Merck Millipore, Burlington, MA, USA) magnetic beads at 4 °C overnight. Immune complexes were extensively washed with lysis buffer, and co-precipitated complexes were analyzed by Western blotting using phospho-ATM/ATR substrate (S*Q) antibody (Cell Signaling, Danvers, MA, USA) detecting phosphorylated SQ motifs (p-SQ).

For nuclear and cytoplasmic extract preparation, the cells were resuspended in five packed cell volume of buffer A containing 20 mM Tris, pH 7.6, 0.1 mM EDTA, 2 mM MgCl_2_, 1 mM PMSF supplemented with 0.2 mM protease inhibitor cocktail (Merck Millipore, Burlington, MA, USA) and phosphatase-inhibitor-mix I (Serva, Heidelberg, Germany). Cells were incubated for 10 min on ice. Thereafter, CHAPS (Merck Millipore, Burlington, MA, USA) was added at a final concentration of 0.6% (*v/v*), and lysates were homogenized by being passed through a 20 G needle for three times. Nuclei were pelleted by centrifugation at 600× *g* for 5 min at 4 °C, and supernatant containing cytoplasmic proteins was collected and stored at −80 °C. The remaining nuclei were washed three times in buffer A containing 0.6% CHAPS. The nucleic pellets were lysed in buffer B containing 20 mM HEPES, pH 7.9, 0.4 M NaCl, 2.5% glycerol, 1 mM EDTA, 1 mM PMSF, 0.5 mM DTT, supplemented with 0.2 mM protease inhibitor cocktail (Merck Millipore, Burlington, MA, USA) and phosphatase-inhibitor-mix I (Serva, Heidelberg, Germany), by repeated freezing and thawing. Supernatants containing soluble nucleic proteins were collected by centrifugation at 20,000× *g* for 20 min and stored at −80 °C.

### 2.9. Quantification of HBc, ATM, Phospho-HBc (p-HBc) and Phospho-ATM (p-ATM)

The intensity of the HBc, ATM, p-HBc or p-ATM signal was calculated as the mean of two to three independent experiments, as specified in figure legends, using Image Studio Lite Software for densitometric analysis, and normalized to the corresponding level of β-actin, total HBc or total-ATM expression, respectively. The levels of p-HBc, p-ATM, HBc or ATM in control cells (untreated cells or at time-point 0 h) were arbitrarily set to 1. Each independent experiment was performed in one replicate.

### 2.10. HBeAg Detection by ELISA

The titers of secreted HBeAg were quantified by ELISA. HepG2-NTCP cell culture supernatants were collected and centrifuged at 120× *g* for 10 min to remove cellular debris, transferred to clean tubes and stored at −80 °C until the antigen measurement. The titers of HBeAg were measured using a commercial ELISA kit (Bioneovan, Beijing, China) according to the manufacturer’s instructions.

### 2.11. Statistical Analysis

Statistical analyses were performed in GraphPad Prism version 9.1.2 (GraphPad Software, San Diego, CA, USA). Results in graphs are expressed as mean and standard deviations. The differences in the mean of p-HBc, p-ATM, HBc or HBeAg between different Etp treatments and the control (ctrl) at various time points were analyzed with a two-tailed paired *t* test, *n* = 3.

## 3. Results

### 3.1. Etoposide- and H_2_O_2_-Induced HBc Phosphorylation

**Figure 1:**
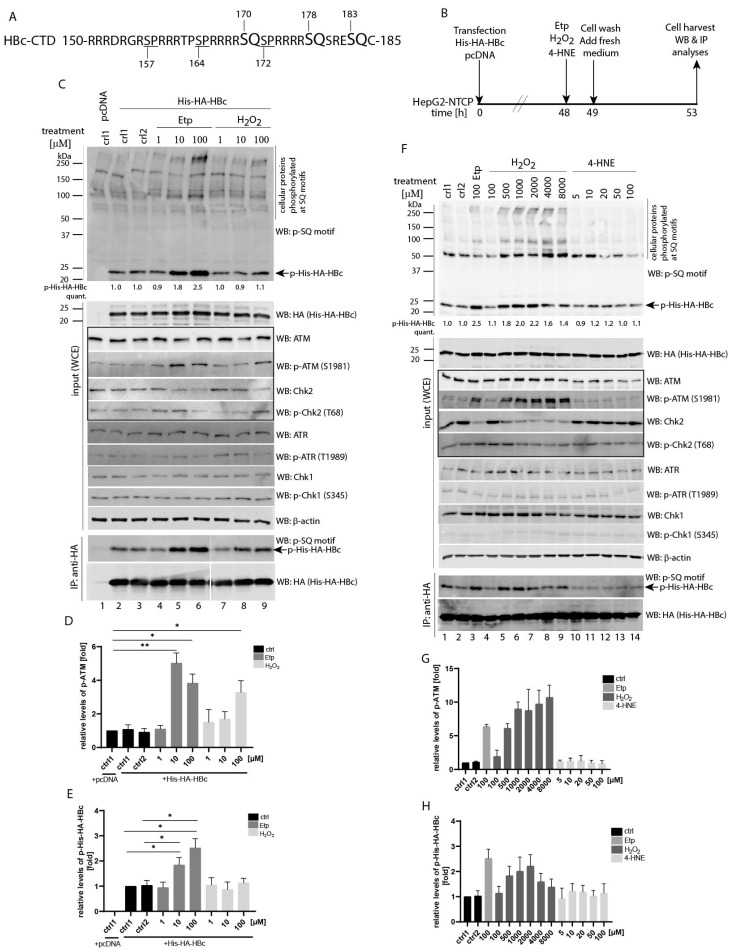
Identification of etoposide- and H_2_O_2_-induced HBc phosphorylation. (**A**) Schematic representation of SP (underlined, S157, S164 and S172) and SQ (larger font, S170, S178 and S183) motifs in the C-terminal domain of the HBc protein. The sequence of wild type (wt) HBc corresponds to a 185-aa HBc of HBV genotype A, subtype adw2. (**B**) The experimental outline: HepG2-NTCP cells were transfected with equal amounts of His-HA-HBc wt or an empty vector (pcDNA). Forty-eight hours after transfection, the cells were treated with etoposide, hydrogen peroxide (H_2_O_2_) or 4-hydroxynonenal (4-HNE) for 1 h. Incubations of cells in medium or vehicle (0.1% DMSO) were labelled as ctrl1 and ctrl2, respectively. After treatment, the cells were washed twice and cultured in DMEM medium supplemented with 10% fetal bovine serum for an additional 4 h. (**C**) Western blot analysis of whole cell extracts (20 µg, marked as input (WCE)) probed with antibodies (from the top to the bottom) against phosphorylated SQ motif (p-SQ), HA, ATM, phospho-ATM (p-ATM), Chk2, phospho-Chk2 (p-Chk2) ATR, phospho-ATR (p-ATR), Chk1, phospho-Chk1 (p-Chk1), and β-actin that was used as a loading control. The ATM-Chk2 pathway that is affected by etoposide treatment is highlighted by the box. The arrows indicate the expected position of the 24 kDa His-HA-HBc protein (21.5 kDa of HBc plus 2.5 kDa of His-HA tag) that is phosphorylated at the SQ sites (p-His-HA-HBc). IP: anti-HA represents the cell lysates (800 µg) precipitated with anti-HA antibodies (His-HA-HBc). The precipitated complexes were analyzed by Western blot with anti-phospho-SQ motif (p-SQ) or anti-HA antibodies. The intensity of the HBc phosphorylation signal (p-His-HA-HBc) was quantified and normalized to the corresponding level of the total His-HA-HBc expression. The level of HBc phosphorylation in untreated control cells (ctrl1) was set to 1. The p-His-HA-HBc values were calculated as mean of three independent experiments. (**D**,**E**) The statistical analyses of the ATM phosphorylation signal (p-ATM; shown in (**D**)) and the HBc phosphorylation signal (p-His-HA-HBc; shown in (**E**)) that were determined in (**C**) using Image Studio Lite Software for densitometric analysis. The data are shown as mean ± SD from three independent experiments. The asterisks indicate statistically significant differences between untreated controls (ctrl1 and ctrl2) and Etp-treated groups determined by a two-tailed paired *t* test, * *p* < 0.05, ** *p* < 0.01, *n* = 3. (**F**) Western blot analyses of whole cell extracts (input (WCE)), and immunoprecipitation (IP:anti-HA) were analyzed as in (**C**) with the only difference that the p-His-HA-HBc values were calculated as mean of two independent experiments. (**G**,**H**) The densitometric analyses of the ATM phosphorylation signal (p-ATM; shown in (**G**)) and the HBc phosphorylation signal (p-His-HA-HBc; shown in (**H**)) that were determined in (**F**) using Image Studio Lite Software. The data are shown as mean ± SD from two independent experiments.

Several recent studies identified the ATM/ATR pathway as an activator of HBV replication and cccDNA formation. These findings led us to investigate whether activation of ATM and/or ATR kinases may result in phosphorylation of the HBc protein. ATM and ATR share substrate specificity, recognizing Ser-Gln (SQ) and Thr-Gln (TQ) motifs. While there is no TQ motif present in the HBc protein sequence, the C-terminal domain contains three SQ sites involving serine residues at positions 170, 178 and 183 (unless stated otherwise, the positions of amino acids are derived from a 185-aa variant of HBc throughout the study (Figure 1A). This region of HBc is rich in arginine residues, and was previously shown to be frequently modified by phosphorylation.

Since the ATM/ATR pathway can be activated in response to treatment with genotoxic agents, we decided to investigate HBc protein phosphorylation in transfected HepG2-NTCP cells after treatment with various concentrations of etoposide, hydrogen peroxide (H_2_O_2_) or 4-hydroxynonenal (4-HNE) (Figure 1B, experimental outline). It is known that ATM phosphorylates the Thr68 of Chk2 upon DNA damage, whereas ATR phosphorylates the Ser345 of Chk1 at stalled replication forks [36]. As shown in Figure 1C, treatment with etoposide led to increased levels of ATM autophosphorylation at Ser1981, as well as phosphorylation of Chk2 at Thr68. The highest level of ATM phosphorylation was achieved with two etoposide concentrations of 10 and 100 µM (Figure 1D). The immunoblotting analysis, using antibodies against the phospho-ATM/ATR substrate (S*Q) that recognize phospho-SQ (p-SQ) motifs, revealed that the level of HBc phosphorylation (p-His-HA-HBc) (Figure 1C, upper panel, marked by an arrow) was considerably increased after 10 and 100 µM etoposide (1.8- and 2.5-fold increase compared to control group; Western blots were visualized using an LI-COR Odyssey CLx system and the Image Studio Lite Software was used for quantitative densitometric analysis). Upregulated phosphorylation of HBc was further confirmed by immunoprecipitation (Figure 1C, bottom IP:anti-HA panels) and the differences in HBc phosphorylation between etoposide-treated and non-treated samples appeared to be statistically significant (Figure 1E). The used treatment conditions did not result in any activation of ATR-Chk1 as assayed by immunoblot with specific phospho-ATR (p-ATR, Thr1989) or phospho-Chk1 (p-Chk1, Ser345) antibodies. In comparison, H_2_O_2_ treatment at concentration ranging from 1 to 100 µM did not result in increased HBc phosphorylation (Figure 1C, lanes 7–9). When we used higher concentrations of H_2_O_2_ up to 8000 µM (Figure 1F), the levels of phosphorylated ATM (Figure 1G) and phosphorylated HBc (Figure 1H) gradually increased. The phosphorylation of HBc reached its maximum of 2.2-fold increase at 2000 µM H_2_O_2_ (Figure 1F, lane 7 and densitometric quantification in Figure 1H). Notably, treatment with 4-HNE, which was previously used for activation of the ATR-Chk1 pathway [37], did not have any effect on activation of either ATM/ATR, their downstream cellular targets or HBc (Figure 1F, lanes 10–14). Because the highest level of HBc phosphorylation (2.5-fold increase) was induced after treatment with 100 µM of etoposide, we used these conditions in all other subsequent experiments.

### 3.2. Etoposide-Induced Phosphorylation of HBc Is ATM-Dependent

**Figure 2:**
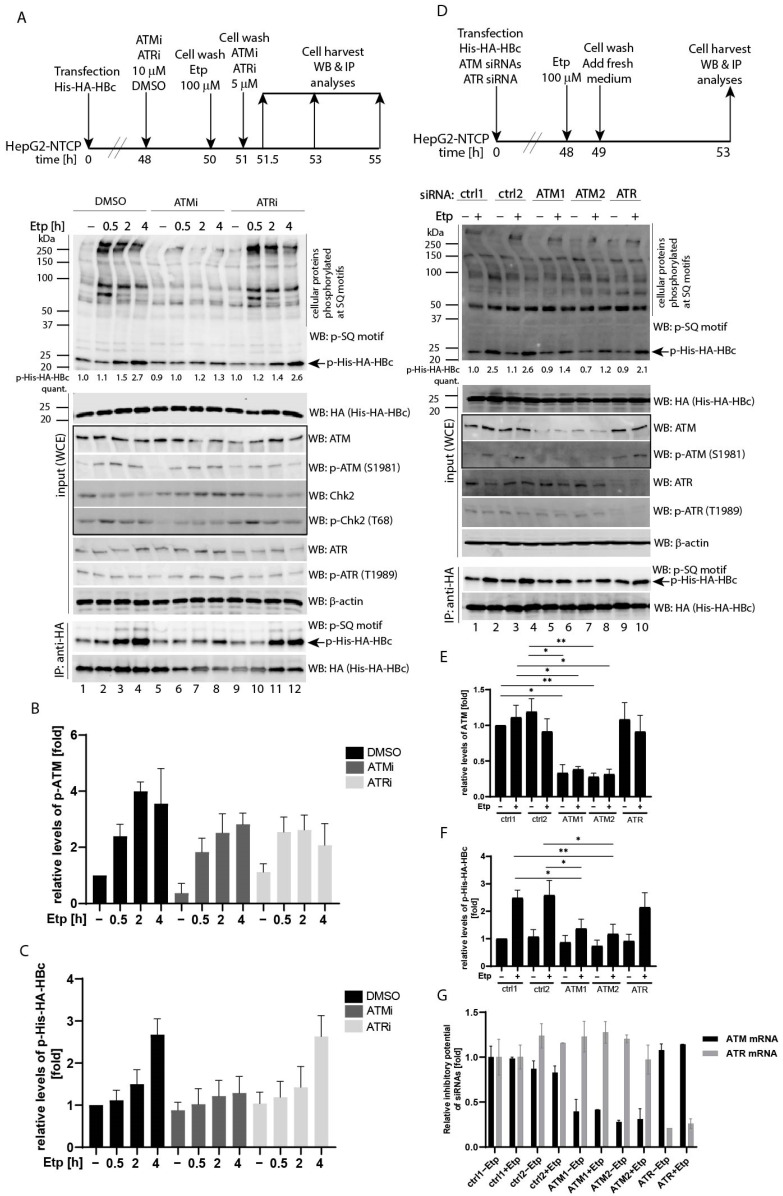
Identification of ATM-Chk2 as a pathway involved in HBc phosphorylation. (**A**) The experimental outline: HepG2-NTCP cells were transfected with His-HA-HBc wt expression plasmid. Forty-eight hours after transfection, the cells were pre-treated with vehicle (DMSO, 0.1%), ATM inhibitor (ATMi—KU55933, 10 μM), or ATR inhibitor (ATRi—VE821, 10 μM) for 2 h, followed by a 1 h etoposide treatment (100 µM). After etoposide treatment, the cells were washed twice, and the ATM/ATR inhibitors (ATMi, ATRi, 5 μM) were re-introduced to the cells and incubated for an additional 0.5, 2, or 4 h. The images display the whole cell extracts (20 µg) that were analyzed by Western blot (marked as input (WCE)) using antibodies (from the top to the bottom) against phosphorylated SQ motif (p-SQ), HA (His-HA-HBc), ATM, p-ATM, Chk2, p-Chk2, ATR, p-ATR, and β-actin (used as a loading control). The ATM-Chk2 pathway that is affected by etoposide treatment is highlighted by box. IP:anti-HA represents the cell lysates (800 µg) that were precipitated (IP) with anti-HA antibodies (His-HA-HBc), and the precipitated complexes were analyzed by Western blot with an anti-phospho-SQ (p-SQ) or with HA antibodies. The arrow indicates the expected position of the 24 kDa His-HA-HBc protein (21.5 kDa of HBc plus 2.5 kDa of His-HA tag) that is phosphorylated at the SQ sites (p-His-HA-HBc). The intensity of the HBc phosphorylation signal (p-His-HA-HBc) was quantified and normalized to the corresponding level of the total His-HA-HBc expression. The level of HBc phosphorylation in untreated control cells (DMSO −) was set to 1. The p-His-HA-HBc values were calculated as mean of two independent experiments. (**B**,**C**) The densitometric analyses of the ATM phosphorylation signal (p-ATM; shown in (**B**)) and the HBc phosphorylation signal (p-His-HA-HBc; shown in (**C**)) that were determined in (**A**) using Image Studio Lite Software. The data are shown as mean ± SD from two independent experiments. (**D**) Downregulation of ATM led to reduced levels of HBc phosphorylation. The experimental outline: HepG2-NTCP cells were transfected with an His-HA-HBc wt expression construct, together with equal amounts of ATM- or ATR-specific siRNAs (ATM1, ATM2, ATR) or non-targeting control siRNAs (ctrl1, ctrl2). Forty-eight hours after transfection, the cells were treated with 100 µM etoposide (+) or 0.1% DMSO (−) for 1 h. After treatment, the cells were washed twice and cultured for an additional 4 h. The whole cell extracts (WCE) were analyzed by Western blot and immunoprecipitation, as in (**A**). The intensity of the HBc phosphorylation signal (p-His-HA-HBc) was quantified and normalized to the corresponding level of the total His-HA-HBc expression. The level of HBc phosphorylation in untreated cells transfected with ctrl1 siRNA (ctrl1 −) was set to 1. The p-His-HA-HBc values were calculated as mean of three independent experiments. (**E**,**F**) The statistical analyses of the ATM protein levels (shown in (**E**)) and the HBc phosphorylation signal (p-His-HA-HBc; shown in (**F**)) that were determined in (**D**) using Image Studio Lite Software for densitometric analysis. The data are shown as mean ± SD from three independent experiments. The asterisks indicate statistically significant differences between ctrl siRNA- (ctrl1 and ctrl2) and ATM siRNA-transfected groups determined by a two-tailed paired *t* test, * *p* < 0.05, ** *p* < 0.01, *n* = 3. (**G**) Down-regulation of endogenous ATM and ATR mRNA expression in siRNA-transfected HepG2-NTCP cells was estimated two days post-transfection by RT-qPCR using primers specific for ATM and ATR cDNAs, and subsequently normalized to the expression of HPRT1. Data is shown as mean ± standard deviation for two independent experiments.

To confirm the role of the ATM pathway in HBc phosphorylation, we assessed the HBc phosphorylation in the presence of ATM/ATR inhibitors and siRNAs (Figure 2). Although etoposide-induced phosphorylation of cellular ATM/ATR substrates, including Chk2, was strongly repressed by the ATM-specific inhibitor (ATMi), KU55933, the autophosphorylation of ATM was not affected by this treatment (Figure 2A, lanes 6–8 and Figure 2B). Treatment of cells with ATMi effectively blocked the etoposide-induced HBc phosphorylation (Figure 2A, p-His-HA-HBc). While in control cells, the etoposide treatment resulted in gradual increase of the HBc phosphorylation, reaching 2.7-fold increase at 4 h post-etoposide treatment, in ATMi-treated cells the levels of phosphorylated HBc were not affected by etoposide (Figure 2C). Inhibition of ATR using the ATR-specific inhibitor IV, VE821 (ATRi) did not produce any effect on phosphorylation of either cellular substrates or HBc. Due to the fact that the use of ATM or ATR inhibitors did not provide clear data regarding the pathway that could be involved in etoposide-induced HBc phosphorylation, we determined the HBc phosphorylation upon depletion of endogenous ATM or ATR (Figure 2D). Transfection of two ATM-specific siRNAs efficiently downregulated ATM expression (Figure 2E) as well as blocked etoposide-induced HBc phosphorylation (2.5- and 2.6-fold increase in control cells vs. 1.4- and 1.2-fold increase in cells with ATM-specific knock-down; Figure 2D, lanes 6 and 8, and Figure 2F). Although the transfection of ATR-specific siRNA resulted in reduced levels of ATR protein, it did not lead to considerable downregulation of phosphorylation of either cellular substrates or HBc. The specificity and efficiency of siRNAs used in the study were also tested using an RT-qPCR approach, and the results are summarized in Figure 2G. Based on the obtained data using ATM-specific siRNAs, we concluded that the activated ATM pathway positively regulates HBc protein phosphorylation. Because the etoposide-, H_2_O_2_-, or 4-HNE-treatment did not activate ATR-Chk1 pathway in these cells under the given experimental settings, the possible role of ATR in HBc phosphorylation cannot be either confirmed or excluded on the basis of these results.

### 3.3. Mapping of Etoposide-Induced Phosphorylation Sites on HBc

**Figure 3:**
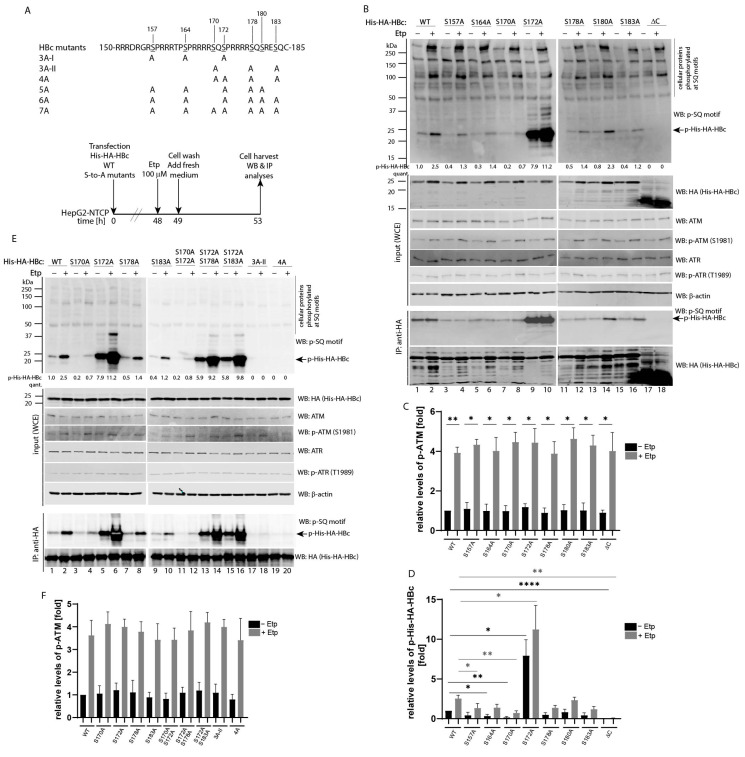

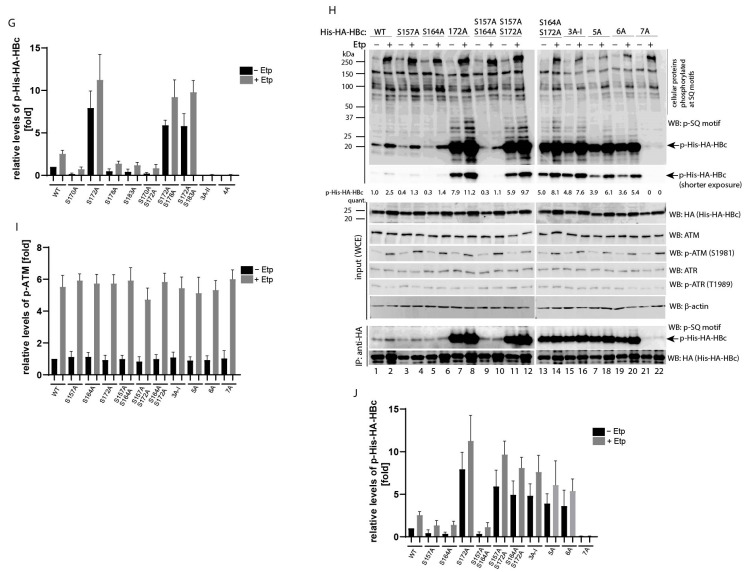
Analysis of etoposide-induced phosphorylation of HBc S-to-A mutants. (**A**) Schematic representation of serine-to-alanine mutations in the C-terminal domain of HBc protein and the experimental outline. The sequence of wild type HBc corresponds to HBV genotype A, subtype adw2. The experimental outline was as follows: HepG2-NTCP cells were transfected with equal amounts of His-HA-HBc wt or S-to-A mutants. Forty-eight hours after transfection, cells were treated with 0.1% DMSO (−) or 100 μM etoposide (+) for 1 h, then washed twice and cultured for an additional 4 h. The isolated whole cell extracts (WCE) were analyzed by Western blot or immunoprecipitation. (**B**) Analysis of serine phosphorylation at SQ motifs of HBc wild type and single S-to-A mutants. The images display the protein lysates (20 µg) that were analyzed by Western blot (marked as input (WCE)) using antibodies (from the top to the bottom) against a phospho-SQ motif (p-SQ), HA (His-HA-HBc), ATM, p-ATM, ATR, p-ATR and β-actin (used as a loading control). IP:anti-HA represents the cell lysates (800 µg) that were precipitated (IP) with anti-HA antibodies (His-HA-HBc), and the precipitated complexes were analyzed by Western blot with an anti-p-SQ or anti-HA antibodies. The arrows indicate the expected position of the 24 kDa His-HA-HBc protein (21.5 kDa of HBc plus 2.5 kDa of His-HA tag) that is phosphorylated at the SQ sites (p-His-HA-HBc). The Western blots are representative of three independent transfection experiments. The intensity of the HBc phosphorylation signal (p-His-HA-HBc) was quantified and normalized to the corresponding level of the total His-HA-HBc expression. The level of HBc wt phosphorylation in untreated cells was set to 1. (**C**,**D**) The statistical analyses of the ATM phosphorylation signal (p-ATM; shown in (**C**)) and the HBc phosphorylation signal (p-His-HA-HBc; shown in (**D**)) that were determined in (**B**) using Image Studio Lite Software for densitometric analysis. The data are shown as mean ± SD from three independent experiments. The asterisks indicate statistically significant differences between HBc wt and HBc mutant groups determined by a two-tailed paired t test, * *p* < 0.05, ** *p* < 0.01, **** *p* < 0.0001, *n* = 3. (**E**) Analysis of serine phosphorylation at SQ motifs of HBc wild type and single or multiple S-to-A mutants as indicated. The Western blot analyses of whole cell extracts (input (WCE)), and immunoprecipitation (IP:anti-HA) were analyzed as in (**B**) with the only difference that the p-His-HA-HBc values were calculated as mean of two independent experiments. (**F**,**G**) The densitometric analyses of the ATM phosphorylation signal (p-ATM; shown in (**F**)) and the HBc phosphorylation signal (p-His-HA-HBc; shown in (**G**)) that were determined in (**E**) using Image Studio Lite Software. The data are shown as mean ± SD from two independent experiments. (**H**) Analysis of serine phosphorylation at SQ motifs of HBc wild type and single or multiple S-to-A mutants. The Western blot analyses of whole cell extracts (input (WCE)), and immunoprecipitation (IP:anti-HA) were analyzed as in (**B**) with the only difference that the p-His-HA-HBc values were calculated as mean of two independent experiments. (**I**,**J**) The densitometric analyses of the ATM phosphorylation signal (p-ATM; shown in (**I**)) and the HBc phosphorylation signal (p-His-HA-HBc; shown in (**J**)) that were determined in (**H**) using Image Studio Lite Software. The data are shown as mean ± SD from two independent experiments.

To further map etoposide-induced phosphorylation sites on HBc, a series of S-to-A substitutions were introduced on each serine residue located in the C-terminal domain, including three SQ and three SP motifs (Figure 3A). Single S-to-A mutations and a ΔC deletion mutant (1–149 aa) lacking the entire C-terminal domain, were introduced into HepG2-NTCP cells and treated with 100 µM etoposide. The levels of ATM and HBc phosphorylation were examined by Western blot and immunoprecipitation (Figure 3B) and quantified using the Image Studio Lite Software for quantitative densitometric analysis (Figure 3C,D). Whereas substitution of Ser180 did not show a significant reduction of phosphorylation, the substitutions at all three SQ sites, including Ser170, Ser178 and Ser183, led to reduced levels of phosphorylation, with the significant degree of reduction observed for the Ser170Ala mutant (statistical analysis in Figure 3D). Interestingly, the mutations of two out of three SP sites involving Ser157 and Ser164 also resulted in decreased phosphorylation at SQ sites. A similar phenomenon has been previously observed, suggesting that priming phosphorylation at the so-called “major” phosphorylation sites at SP motifs may be required for efficient phosphorylation at “minor” SQ sites. Surprisingly, the mutation at Ser172 resulted in massive phosphorylation of HBc in uninduced as well as etoposide-induced cells (Figure 3B, lanes 9 and 10).

To determine which Ser residue is predominantly phosphorylated in a S172A mutant, we transfected HepG2-NTCP cells with different multiple S-to-A mutations and analyzed their level of phosphorylation after activation of ATM pathway using etoposide treatment (Figure 3E,F). Although the mutations of Ser178 and Ser183 only marginally affected phosphorylation of a S172A mutant (Figure 3E, lanes 13–16), the mutation of Ser170 significantly reduced phosphorylation (Figure 3E, lanes 11 and 12 and densitometric analysis in Figure 3G). These data suggested that blocking phosphorylation at the SP site involving Ser172 led to massive phosphorylation of the adjacent SQ site involving Ser170. In addition, the mutation of all three SQ sites (mutant 3A-II) completely abolished etoposide-induced phosphorylation (Figure 3E, lanes 17 and 18).

To further confirm the role of prior phosphorylation at “major” SP sites, we analyzed the level of ATM-induced phosphorylation of various HBc constructs carrying mutations at positions S157 and S164 (Figure 3H and densitometric analysis of p-ATM in Figure 3I). The mutations of Ser157 and Ser164 reduced phosphorylation at the SQ sites of both HBc wild type as well as its S172A mutant (Figure 3H, lanes 3–6 and 11–14 and densitometric analysis of p-His-HA-HBc in Figure 3J). Interestingly, the mutant 6A, which contains six of the seven serine residues mutated to Ala, still exhibited a relatively high level of phosphorylation that was completely abolished after the introduction of the S170A mutation as seen in the mutant 7A (Figure 3H, lanes 19–22 and Figure 3J).

### 3.4. Etoposide-Induced Phosphorylation Occurs on Cytoplasmic HBc

**Figure 4:**
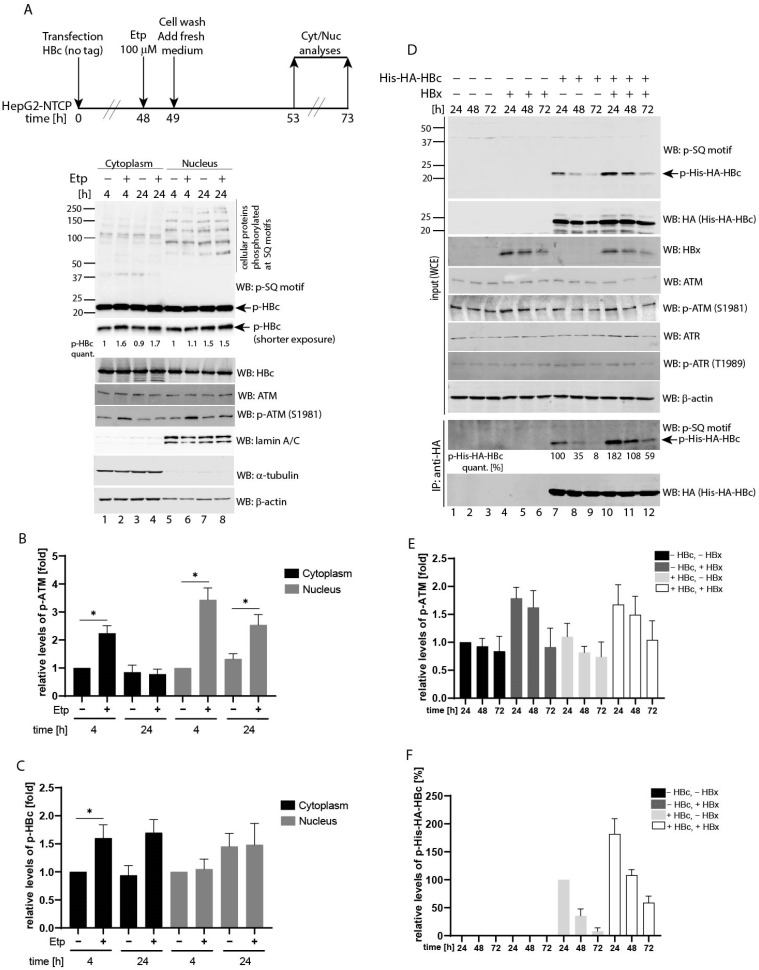
(**A**) Etoposide-induced phosphorylation of HBc occurs in the cytoplasm. The experimental outline: HepG2-NTCP cells were transfected with untagged HBc, followed by 1 h treatment with 100 µM etoposide. 4 and 24 h after treatment, the cells were fractionated into cytoplasmic (left) and nuclear (right) extracts. The protein lysates (10 μg) were analyzed by Western blotting with antibodies (from the top to the bottom) against a phospho-SQ motif (p-SQ), HBc, ATM, p-ATM, lamin A/C (nuclear marker), α-tubulin (cytoplasmic marker) and β-actin (loading control). The arrows indicate the expected position (21.5 kDa) of the HBc protein that is phosphorylated at the SQ sites (p-HBc). (**B**,**C**) The intensity of the ATM phosphorylation signal (**B**) and the HBc phosphorylation signal (**C**), that were determined in (**A**), were quantified using the Image Studio Lite software for densitometric analysis and normalized to the corresponding level of the total ATM or HBc expression. The levels of ATM or HBc phosphorylation in non-treated cells at a 4 h time-point were arbitrarily set to 1. The asterisks indicate statistically significant differences between the control (untreated) and Etp-treated groups determined by a two-tailed paired t test, * *p* < 0.05, *n* = 3. Error bars represent SD of three independent experiments. (**D**) The expression of HBx induced phosphorylation of HBc at the SQ sites. HepG2-NTCP cells were transfected with HBx, together with HBc expression plasmids, as indicated. The images display the cell lysates (20 µg) that were isolated at different time-points post-transfection (24, 48 and 72 h), and analyzed by Western blot (marked as input) using antibodies (from the top to the bottom) against a phospho-SQ motif (p-SQ), HA (His-HA-HBc), HBx, ATM, p-ATM, ATR, p-ATR and β-actin (loading control). The arrow indicates the expected position of the 24 kDa His-HA-HBc protein (21.5 kDa of HBc plus 2.5 kDa of His-HA tag) that is phosphorylated at the SQ sites (p-His-HA-HBc). IP:anti-HA represents the cell lysates (800 µg) that were precipitated with anti-HA antibodies (His-HA-HBc), and the precipitated complexes were analyzed by Western blot with an anti-p-SQ or anti-HA antibodies. The intensity of the HBc phosphorylation signal was quantified and normalized to the corresponding level of the total HBc expression. The level of HBc phosphorylation in the absence of the HBx expression at 24 h post-transfection was set to 100%. The p-His-HA-HBc values were calculated as mean of two independent experiments. (**E**,**F**) The densitometric analyses of the ATM phosphorylation signal (p-ATM; shown in (**E**)) and the HBc phosphorylation signal (p-His-HA-HBc; shown in (**F**)) that were determined in (**D**) using Image Studio Lite Software. The data are shown as mean ± SD from two independent experiments.

Having established that HBc phosphorylation is regulated by activated ATM, we investigated in which cellular compartment the HBc phosphorylation takes place. We performed a cell fractionation assay of HBc-expressing HepG2-NTCP cells. To exclude the possibility that the His-HA tag itself may have some effect on the subcellular localization of HBc, the HepG2-NTCP cells were transfected with HBc expression plasmid without a tag, followed by 1 h treatment with 100 µM etoposide. Four and 24 h post-treatment, the nuclear and cytoplasmic extracts were prepared and analyzed by Western blotting (Figure 4A). The ATM was predominantly activated in the nuclei of etoposide-treated cells as determined by the levels of ATM phosphorylation at Ser1981 (Figure 4B). Nevertheless, some phosphorylated ATM protein could also be detected in the cytoplasm of etoposide-treated cells, but to a much lesser extent. Although the nuclear protein fraction contained phosphorylated HBc, its levels remained unchanged regardless of culture conditions (Figure 4C). Conversely, in the cytoplasm, the pool of phosphorylated HBc increased in etoposide-treated cells. As shown in Figure 4A,C, at 4 and 24 h post-treatment, the cells contained 1.6- and 1.7-fold higher levels of phosphorylated HBc compared to untreated controls. Western blotting with antibodies against alpha-tubulin and lamin A/C were used to assess the purity and quality of cytoplasmic and nuclear extracts preparation, respectively.

### 3.5. Co-Expression of HBV X (HBx) Leads to Upregulated HBc Phosphorylation at SQ Motifs

The cytoplasmic HBx protein was previously shown to induce reactive oxygen species (ROS) production that led to the accumulation of γ-H2AX foci and increased p-ATM and p-Chk2 levels [33]. Based on this data, the expression of HBx could potentially cause cytoplasmic HBc phosphorylation. To test this hypothesis, we transfected the HBx and HBc expressing plasmids into HepG2-NTCP cells and determined the levels of phosphorylated ATM (p-ATM), ATR (p-ATR) and HBc (p-His-HA-HBc) proteins. In agreement with previous studies, transfection of HBx led to an increased ATM phosphorylation, particularly at 24 h post-transfection (Figure 4D, lanes 4 and 10 and densitometric analysis in Figure 4E). Notably, the HBc-expressing cells that were co-transfected with HBx exhibited considerably higher levels of HBc phosphorylation compared to control cells (Figure 4D, lanes 10–12 vs. 7–9 and densitometric analysis in Figure 4F). Thus, an HBx-mediated increase of p-ATM correlated well with elevated levels of phosphorylated HBc. The relative phosphorylation levels of HBc in control cells continuously decreased from 100% to 8% at 24 and 72 h, respectively (Figure 4D, line p-His-HA-HBc quant., and Figure 4F). Interestingly, the levels of p-His-HA-HBc in HBx-expressing cells were almost two-fold higher than in control cells (182% vs. 100%), and phosphorylation decreased at a considerably slower rate. The phosphorylation levels of HBc at 72 h post-transfection were 59% in HBx-expressing cells compared to 8% in HBx-negative control cells.

### 3.6. Effect of Etoposide Treatment on HBV Infection

**Figure 5:**
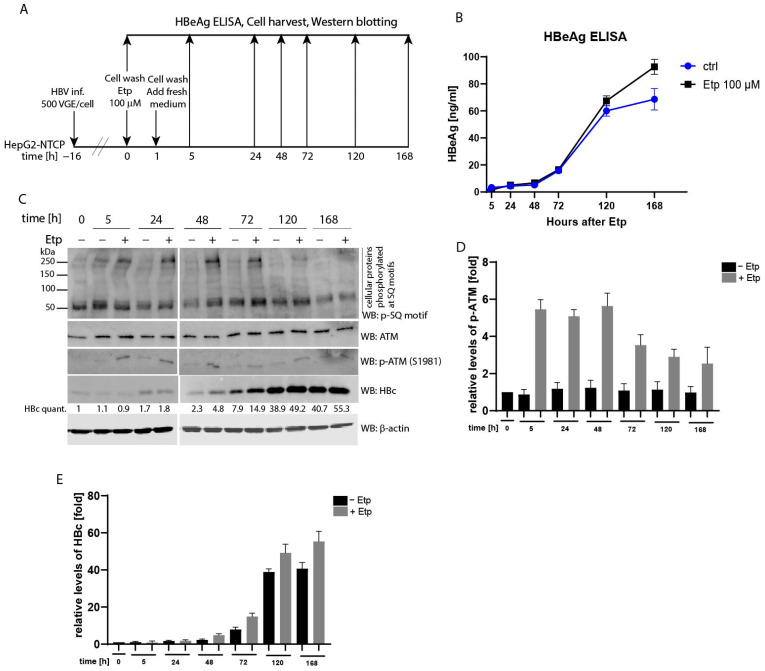
Etoposide treatment of HBV-infected HepG2-NTCP cells stimulates HBV replication. (**A**) The experimental outline: HepG2-NTCP cells were infected with HBV (MOI 500 VGE per cell) and treated with 100 µM etoposide (+) for 1 h, or left untreated (−). At various time-points (0, 5, 24, 48, 72, 120 and 168 h) after addition of etoposide, the levels of HBeAg (**B**) in the culture supernatants were analyzed by enzyme-linked immunosorbent assay (ELISA). Data is shown as mean ± standard deviation for two independent experiments. (**C**) The protein lysates (20 µg) were isolated at different time-points and were analyzed by Western blot using antibodies against a phospho-SQ motif (p-SQ), HBc, ATM, p-ATM and β-actin (loading control). The intensity of the HBc protein signal was quantified and normalized to the corresponding level of β-actin. The amount of the HBc protein in non-treated control cells at time-point 0 h was set to 1. The HBc quant. values were calculated as mean of two independent infection experiments. (**D**,**E**) The densitometric analyses of the ATM phosphorylation signal (p-ATM; shown in (**D**)) and the HBc protein levels (shown in (**E**)) that were determined in (**C**) using Image Studio Lite Software. The data are shown as mean ± SD from two independent experiments.

Several recent studies underlined the importance of the ATM/ATR pathway in supporting HBV replication or formation of cccDNA in the nuclei of infected cells. Kostyusheva et al. [34] demonstrated that transcriptional activation of ATM and ATR led to elevated levels of HBV RNAs and DNA. Likewise, the treatment of cells with DNA-damaging agents, e.g., H_2_O_2_ or doxorubicin, resulted in upregulation of HBV replication that was assayed on the level of HBV RNAs, intracellular or secreted HBV DNA and the HBs antigen. Therefore, we decided to determine whether etoposide treatment may have any effect on the HBc protein and its phosphorylation levels and/or secretion of the HBe antigen (HBeAg) in HBV-infected cells. To test this, HepG2-NTCP cells were infected with HBV (MOI 500 viral genome equivalents (VGE) per cell) and subsequently treated with 100 µM etoposide for 1 h (Figure 5A). HBV replication was evaluated both on the level of secreted HBe analyzed by ELISA (Figure 5B), and on the level of HBc in protein lysates analyzed by Western blotting probed with anti-HBc antibody (Figure 5C). The data were collected at several time-points of 0, 5, 24, 48, 72, 120, and 168 h, where time 0 represents the start of etoposide treatment (Figure 5A, experimental outline). As shown in Figure 5B, at early time-points, from 0 to 48 h, the rate of HBV infection was similar for both etoposide-treated and non-treated samples. At 72 h, the difference started to become more apparent and at later times, including 120 and 168 h, there was a considerably higher level of HBe in the media of etoposide-treated cells compared to untreated controls. The Western blot analysis of cellular lysates detected higher levels of both phosphorylated ATM and intracellular HBc protein in HBV-infected cells treated with etoposide than in non-treated controls (Figure 5C and densitometric analysis in Figure 5D,E). Although at early time-points the relative levels of HBc were very low and did not differ greatly between etoposide-treated and non-treated controls, the differences in HBc levels started to appear at later time-points. From 48 to 168 h, the relative levels of HBc ranged from 2.3- to 40.7-fold in non-treated controls compared to 4.8- and 55.3-fold in etoposide-treated samples (Figure 5E). We also attempted to examine the level of etoposide-induced HBc phosphorylation in these cells, but due to very low levels of HBc protein, especially at earlier time-points, HBc phosphorylation was below the detection limit of Western blotting (data not shown).

**Figure 6:**
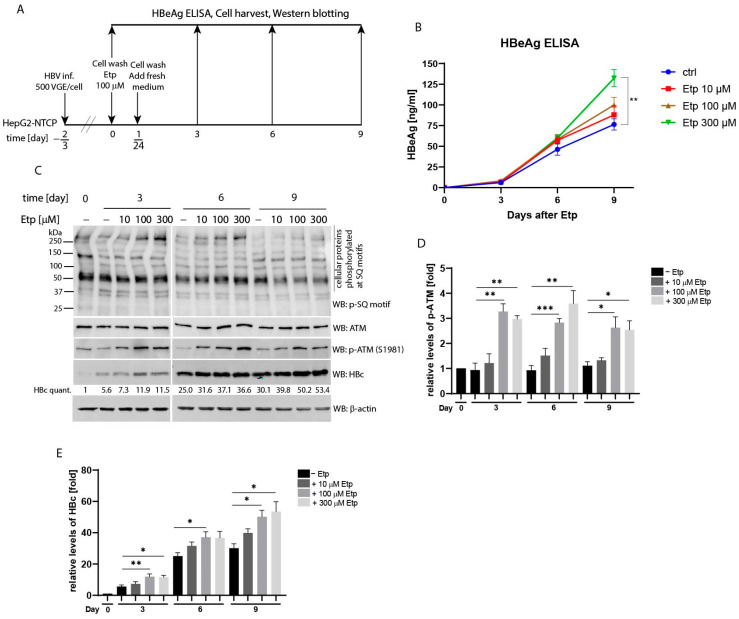
The stimulatory effect of etoposide treatment on the level of HBeAg and HBc is dose-dependent. (**A**) The experimental outline: HepG2-NTCP cells were infected with HBV (MOI 500 VGE per cell) and treated with 10, 100 or 300 µM etoposide for 1 h, or left untreated (−). At various time-points (0, 3, 6 and 9 days) after addition of etoposide, the levels of HBeAg (**B**) in the culture supernatants were analyzed by an enzyme-linked immunosorbent assay (ELISA). Data is shown as mean ± standard deviation for three independent experiments. ** *p* < 0.01 obtained by a two-tailed paired *t* test, *n* = 3. (**C**) The protein lysates (20 µg) isolated at different time-points were analyzed by Western blot using antibodies against a phospho-SQ motif (p-SQ), HBc, ATM, p-ATM and β-actin (loading control). The intensity of the HBc protein signal was quantified and normalized to the corresponding level of β -actin. The amount of the HBc protein in non-treated control cells at time-point of 0 day was set to 1. The HBc quant. values were calculated as mean of three independent infection experiments. (**D**,**E**) The statistical analyses of the ATM phosphorylation signal (p-ATM; shown in (**D**)) and the HBc protein level (shown in (**E**)) that were determined in (**C**) using Image Studio Lite Software for densitometric analysis. The data are shown as mean ± SD from three independent experiments. The asterisks indicate statistically significant differences between untreated controls (marked as −) and Etp-treated groups determined by a two-tailed paired *t* test, * *p* < 0.05, ** *p* < 0.01, *** *p* < 0.001, *n* = 3.

Next, we investigated whether the effect of etoposide treatment on secretion of the HBe antigen as well as expression of the HBc protein, is concentration dependent. To do this, we infected HepG2-NTCP cells with HBV (MOI 500 VGE per cell) and subsequently treated them with different concentrations of etoposide ranging from 10 to 300 µM (Figure 6A). The levels of secreted HBe in culture supernatants (Figure 6B) and intracellular HBc protein (Figure 6C) were thereafter determined at 0, 3, 6, and 9 days post-etoposide treatment. As shown in Figure 6B, the quantity of secreted HBe gradually increased after etoposide treatment in a dose-dependent manner. At the final time-point of 9 days, the levels of HBe in supernatants of cells treated with 300 µM etoposide were significantly increased compared to the untreated controls. The Western blot analysis of cellular lysates detected significantly upregulated levels of phosphorylated ATM (p-ATM) in cells treated with 100 and 300 µM etoposide (Figure 6C and statistical analysis in Figure 6D). Likewise, the relative levels of intracellular HBc (Figure 6C) were also elevated in etoposide-treated cells with maximum levels reached at day 9 after treatment with 300 µM etoposide (30.1-fold in control cells vs. 53.4-fold in etoposide-treated cells; Figure 6E).

**Figure 7:**
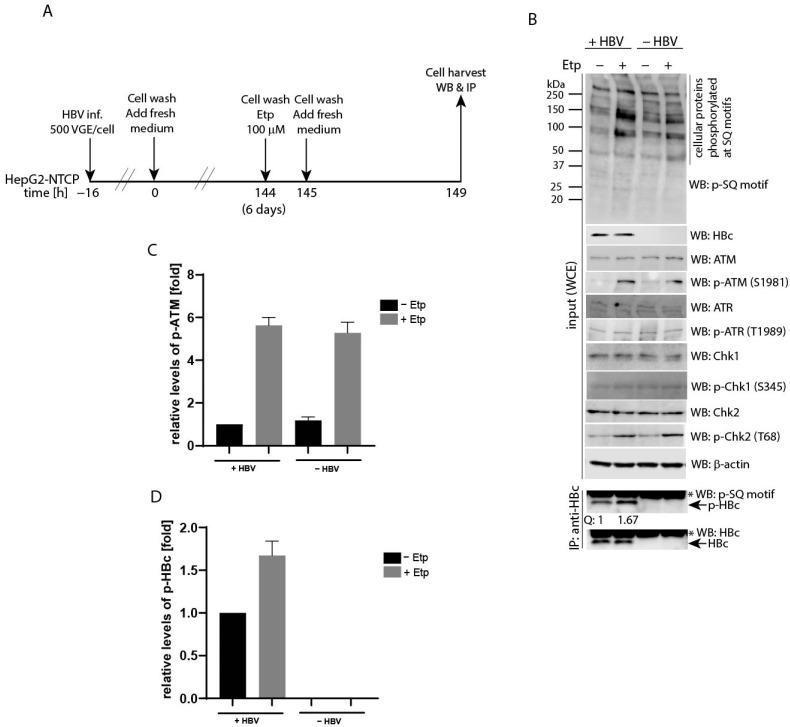
Etoposide treatment upregulates phosphorylation of the HBc protein in HBV-infected cells. (**A**) Experimental outline: HepG2-NTCP cells were infected with HBV (MOI 500 VGE per cell) or left uninfected. Six days post-infection, the cells were treated with 100 µM etoposide (+) for 1 h, or left untreated (0.1% DMSO, marked as −). After treatment, the cells were washed twice and cultured for an additional 4 h. (**B**) The isolated protein lysates (30 µg) were analyzed by Western blot (input) with antibodies (from the top to the bottom) against a phospho-SQ motif (p-SQ), HBc, ATM, p-ATM, ATR, p-ATR, Chk1, p-Chk1, Chk2, p-Chk2 and β-actin (loading control). IP:anti-HBc represents the cell lysates (1 mg) that were immunoprecipitated with anti-HBc, and the precipitated complexes were analyzed by Western blot with an anti-phospho-SQ motif (p-SQ) or anti-HBc (two bottom panels). The intensity of the HBc phosphorylation signal was quantified (line Q:) and normalized to the level of precipitated HBc. The level of HBc phosphorylation in non-treated cells was set to 1. The p-HBc values were calculated as mean of two independent experiments. The asterisks indicate a non-specific IgG signal. (**C**,**D**) The densitometric analyses of the ATM phosphorylation signal (p-ATM; shown in (**C**)) and the HBc phosphorylation signal (shown in (**D**)) that were determined in (**B**) using Image Studio Lite Software. The data are shown as mean ± SD from two independent experiments.

To analyze ATM-regulated phosphorylation of HBc in HBV-infected hepatocytes, HepG2-NTCP cells were infected with HBV (MOI 500 VGE per cell), followed by 1-h etoposide treatment 6 days after infection (Figure 7A). The levels of phosphorylated HBc protein were determined by immunoprecipitation using specific anti-HBc antibodies, followed by Western blot with anti-phospho-SQ motif (p-SQ) antibodies. As shown in Figure 7B, the etoposide-treated cells exhibited higher levels of phosphorylated cellular substrates, p-ATM, p-Chk2 as well as p-HBc, compared to untreated controls. The relative levels of phosphorylated ATM (p-ATM; Figure 7C) and phosphorylated HBc (p-HBc; Figure 7D) were approximately 5- and 1.67-fold higher, respectively, after etoposide-induced activation of the ATM-Chk2 pathway. The levels of phosphorylated ATR and Chk1 remained unaffected.

## 4. Discussion

Many DNA viruses rely on the host DDR pathway for their efficient propagation. Viruses take advantage of the DDR to modulate the cell cycle and hijack cellular proteins to support viral replication [38,39,40]. In fact, the lytic replication of DNA viruses, including EBV, KSHV or HSV, almost uniformly require activated ATM/ATR [41].

### 4.1. ATM-Dependent Phosphorylation of HBc at S170

Growing evidence that the Hepatitis B virus relies on activated DDR for its effective replication prompted us to study the interaction between the host ATM/ATR pathway and viral proteins. In this study, we investigated the role of activated ATM/ATR in phosphorylation of the HBc protein. Our presented data are the first to identify HBc as the phosphorylation target regulated by ATM-Chk2 after the induction of the DNA damage response pathway by etoposide or H_2_O_2_ treatment. Importantly, the down-regulation of ATM using ATM-specific siRNAs led to substantially reduced levels of HBc phosphorylation. Thus, this study revealed that ATM is likely to be the major kinase responsible for the etoposide-induced HBc phosphorylation. Surprisingly, none of the genotoxic drugs used in this study could activate the ATR-Chk1 pathway. Although H_2_O_2_ was previously shown to trigger the activation of Chk1 independently of ATM, and H_2_O_2_ treatment was successfully used in other HBV study using HepG2 cell line [34], we were not able to detect either autophosphorylation of ATR at T1989 or the phosphorylation of its substrate Chk1 at S345. Likewise, the treatment of cells with ATR-specific inhibitor or transfection of ATR-siRNA did not display any effect on HBc phosphorylation. Thus, based on presented data, the activation of ATM-Chk2 leads to elevated HBc phosphorylation at SQ motifs. Nevertheless, the potential role of ATR in this process still remains to be addressed.

The mutation analysis identified the serine residues that were modified by ATM-mediated phosphorylation. All three serines, S170, S178 and S183 (S168, S176 and S181 in a 183-aa HBc variant), that were part of SQ motifs located in the CTD were phosphorylated. Notably, the S-to-A mutation involving S170 displayed the highest reduction in phosphorylation, suggesting that this serine is predominantly modified by activated ATM kinase. As reported previously, the majority of wt HBc expressed in human cells is hyperphosphorylated that could be detected by phos-tag SDS PAGE as two slowly migrating species [19,42]. Such extensive constitutive phosphorylation of wt HBc may be the reason, why etoposide-induced activation of ATM-Chk2 pathway led to relatively low increase in HBc phosphorylation (approximately 2.5-fold increase in etoposide-treated cells compared to untreated controls).

### 4.2. The Role of “Major” Phosphorylation Sites in HBc Phosphorylation at SQ Motifs

Furthermore, we showed that efficient ATM-mediated phosphorylation of HBc requires prior phosphorylation at S157 and S164. Similar phenomenon was described previously for Plk1 and HBc [22], indicating that the priming phosphorylation event on two major serine residues is required for an efficient phosphorylation process at minor phosphorylation sites. Surprisingly, the S-to-A mutation at position 172 (S170 in a 183-aa HBc variant) led to massively upregulated phosphorylation of adjacent serine residue 170 in non-treated as well as etoposide-treated cells. Similar observation was reported recently by Xi et al. [42] who showed that blocking the phosphorylation of major SP sites in a 3A mutant led to dramatically increased phosphorylation of SQ sites while decreasing the phosphorylation at S180 (S178 in a 183-aa HBc variant of HBc). This enormous phosphorylation could be a consequence of either some kind of compensatory mechanism or competition between phosphorylation of two closely adjacent phospho-acceptor sites. Notably, Ser170 was recently described as a phosphorylation site that is vital for HBV replication and that contributes to efficient pgRNA encapsidation and rcDNA synthesis [17].

### 4.3. Phosphorylation of HBc CTD and Its Role in HBV Life Cycle

Dynamic phosphorylation and dephosphorylation of HBc CTD and cross-talk between phosphorylation sites are important mechanisms involved in various steps of HBV replication, including pgRNA packaging, reverse transcription, nucleocapsid (NC) formation and virion maturation [11,15,17,19,43,44,45,46]. The charge balance hypothesis highlights the importance of balanced electro-static interactions between the positively charged arginine-rich CTD of HBc and the negatively charged encapsidated nucleic acids [47,48,49]. Thus, modulation of the negative charges derived from phosphoserines, allows HBV to maintain electrostatic homeostasis. HBc dimers in infected cells are hyperphosphorylated, but encapsidation of pgRNA, reverse transcription and NC maturation require dephosphorylation [15,43,50]. The process of dephosphorylation counterbalances the increasing negative charge inside the nucleocapsid as a result of conversion of pgRNA to rcDNA. On the other hand, release of rcDNA from NCs for subsequent cccDNA formation requires re-phosphorylation of HBc [20,51]. The process of phosphorylation and dephosphorylation is tightly regulated by cell kinases and phosphatases. Two cellular phosphatases, protein phosphatase 1 (PP1) and protein phosphatase 2A (PP2A), were recently shown to be responsible for HBc dephosphorylation [42,52]. PP1 was further shown to be co-packaged with viral pgRNA into nucleocapsids where it catalyzes dephosphorylation of HBc [52]. Several host kinases involved in HBc phosphorylation have been identified till today. Among them, cyclin-dependent kinase 2 (Cdk2), protein kinase A and C (PKA and PKC), serine-arginine protein kinase 1 and 2 (SRPK1/2) and Polo-like kinase 1 (Plk1) [16,22,53,54,55,56].

Plk1 was shown to phosphorylate two out of three HBc SQ sites involving S170 and S178 that were also identified as substrate motifs targeted by ATM in the presented study [22]. Interestingly, treatment of HBV-infected hepatocytes with Plk1-specific inhibitors or transfection of Plk1 siRNAs led to reduced formation/accumulation of HBV nucleocapsids and decreased intracellular accumulation of HBV DNA [22]. It would be of interest to determine whether ATM-regulated phosphorylation of HBc protein may have similar effect on viral replication. Therefore, the functional link between ATM activation and HBc phosphorylation and their impact on HBV infection require additional studies.

### 4.4. The Role of Cytoplasmic ATM Pathway in the Regulation of HBc Phosphorylation

Although the ATM protein was shown to be localized mainly in the nucleus of mitotic cells, a number of studies indicated that ATM was also present in the cytoplasm, including peroxisomes and cytoplasmic vesicles [57,58,59,60]. Moreover, apart from its nuclear role in the DDR, ATM is also activated in the cytosol by ROS and hypoxia, and it can modulate autophagy and mitochondrial biogenesis through multiple molecular mechanisms [61,62]. Importantly, etoposide, a topoisomerase II inhibitor, is known for inducing both ATM-dependent pathways through the induction of DNA double-strand breaks (DSBs) as well as the production of ROS [63,64,65]. Although, etoposide treatment of HepG2-NTCP resulted in induced phosphorylation of both nuclear and cytoplasmic ATM at S1981 (shown in Figure 4A), etoposide-induced phosphorylation of HBc could be detected only in the cytoplasm. This data suggests that the cytoplasm is the primary site for ATM-regulated phosphorylation of HBc, resulting in an increased pool of phosphorylated HBc in this cellular compartment. This notion was also supported by the fact that the expression of HBx that was previously shown to induce ROS production in cells also upregulated HBc phosphorylation. Due to the fact that the HBx expression plasmid used in this study was under the control of CMV promoter, that may cause the HBx overexpression and the induction of non-physiological cellular stress conditions [66], the link between HBx expression and HBc phosphorylation needs to be addressed in the context of HBx-deficient HBV replication.

### 4.5. Interaction between HBV and Host ATM-Regulated Pathway

The effects of DNA damaging agents on HBV replication were well documented by Kostyusheva et al. [34]. The authors showed that treatment of HBV-infected cells with doxorubicin or H_2_O_2_ led to significant upregulation of pgRNA, S-RNA, intracellular and secreted HBV DNA and HBsAg. Here, we extended their findings by showing that etoposide treatment may provide growth advantage to the replicating virus as demonstrated by elevated levels of intracellular HBc protein and secreted HBe antigen. Especially at later time-points after etoposide-treatment, the differences in levels of HBc and HBe became clearly apparent. The possible mechanism of ATM action on HBe and HBc expression could act via the induction of cell cycle arrest leading to increased gene expression from accumulated cccDNA in non-dividing cells. This could be enough to cause relatively weak effect on HBeAg secretion in the infection studies.

From a diagnostic perspective, HBeAg is an important marker of HBV replication. The differences in HBeAg secretion in our study appear to be relatively low (less than 2-fold changes) compared to chronically infected patients where viral reactivation often leads to dramatic upregulation of antigen production. In patients, HBV reactivation and liver disease progression depends on many different factors, that include patient’s age and immunosuppression status, co-infection with other viruses and additional anti-viral or anti-cancer therapies. Therefore, we conclude that even relatively low differences observed in infection studies may have biological significance and become clinically important.

Consistent with our in vitro results, frequent HBV reactivation has been observed in patients with chronic hepatitis after the use of etoposide during different anti-cancer chemotherapies [67,68,69]. In fact, a considerable number of HBV-positive cancer patients develop a drug-related liver toxicity due to reactivation of HBV replication [70]. While acute hepatitis due to the reactivation of HBV replication is a frequent condition in patients undergoing anti-cancer treatment, the reactivation of HCV (Hepatitis C virus) is extremely rare [71].

The importance of the ATM signaling pathway in HBV infection and HBV-associated carcinogenesis was also recently supported by the study of Dansako et al. [72], who demonstrated that HBV infection led to the activation of the ATM-Chk2 signaling pathway in infected cells. Moreover, they proposed the model in which the extracellular vesicles (EVs) derived from HBV-infected cells activated the ATM-Chk2 signaling in naïve cell through intercellular transfer of mitochondrial DNA (mtDNA) from HBV-infected cells to neighboring cells.

In summary, the activation of the ATM pathway leads to upregulation of HBc phosphorylation that occurs on SQ motifs located in the C-terminal domain. Further in-depth analysis will be needed to explore the interaction between HBV and the host ATM pathway and to define the role of ATM-mediated HBc phosphorylation in HBV replication or reactivation.

## 5. Conclusions

The importance of the host DDR pathway in HBV replication and associated pathogenesis has been emerging recently. The detailed understanding of the molecular mechanism and functional outcome of the interaction between HBV and host ATM/ATR will enable future studies that may lead to the design of new therapeutic approaches.

## Data Availability

Data is contained within the article.
